# Life’s Irritabilities

**Published:** 1856-09

**Authors:** 


### LIFE'S IRRITABILITIES.

WHAT'S the use of it? Don't worry yourself to death on account of what other people may say of you, as long as you know it is not true. Take care of the truth, that's your business. All falsehoods go to the bosom of their father the Devil, and their framers soon follow. So much as to falsehoods *of* you. As to falsehoods *to* you, and as to every tale the most remotely prejudicial to another, treat it, and the narrator, with the utmost possible indifference, until you hear the story of the other party; this only is just, and wise, and kind.

